# Impact of Particle Sedimentation in Pendant Drop Tensiometry

**DOI:** 10.1021/acs.langmuir.2c01193

**Published:** 2022-08-09

**Authors:** Roy J. B. M. Delahaije, Leonard M. C. Sagis, Jack Yang

**Affiliations:** † Laboratory of Physics and Physical Chemistry of Foods, Wageningen University, Bornse Weilanden 9, 6708WG Wageningen, The Netherlands; ‡ Laboratory of Biobased Chemistry and Technology, Wageningen University, Bornse Weilanden 9, 6708WG Wageningen, The Netherlands; § FrieslandCampina Innovation Centre, Bronland 20, 6708 WH Wageningen, The Netherlands

## Abstract

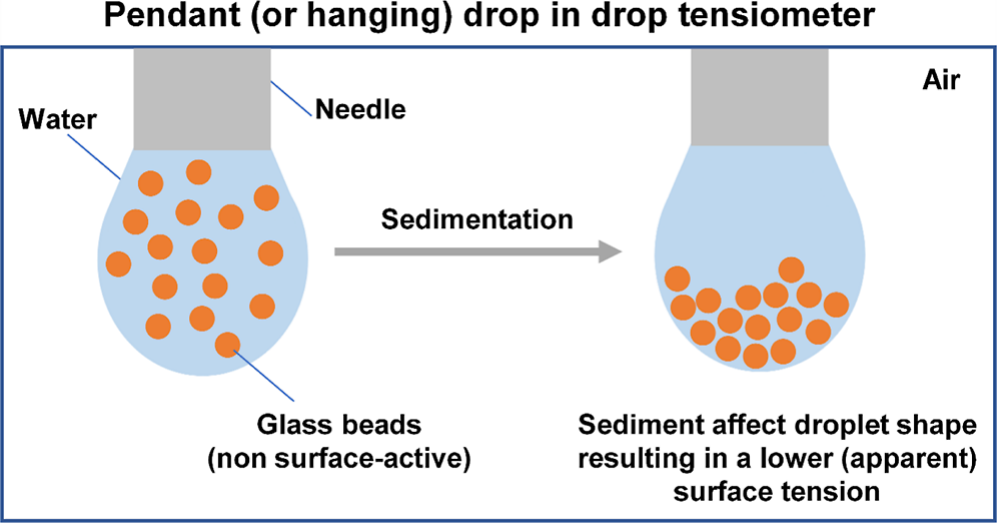

Understanding the interface-stabilizing properties of surface-active components is key in designing stable macroscopic multiphase systems, such as emulsions and foams. When poorly soluble materials are used as an interface stabilizer, the insoluble material may sediment and interfere with the analysis of interfacial properties in pendant (or hanging) drop tensiometry. Here, the impact of sedimentation of particles on the interfacial properties determined by pendant drop tensiometry was evaluated using a model system of whey protein isolate and (non surface-active) glass beads (2.2–34.7 μm). Although the glass beads did not adsorb to the air–water interface, a 1% (w/w) glass bead solution appeared to decrease the surface tension by nearly 12 mN/m after 3 h. A similar effect was shown for a mixture of whey proteins and glass beads: the addition of 1% (w/w) of glass beads led to an apparent surface tension decrease of 31 mN/m rather than the 20 mN/m observed for pure whey proteins. These effects are attributed to the sedimentation of particles near the apex of the droplet, leading to droplet shape changes, which are interpreted as a decrease in surface tension using tensiometer software. The droplet density at the apex increases due to sedimentation, and this density increase is not accounted for when fitting the droplet shape with the Young–Laplace equation. The result is the observed apparent decrease in surface tension. In contrast to the significant impact of sedimenting material on the surface tension measurements, the impact on the results of oscillatory deformations was limited. These findings show that the impact of sedimentation should be considered when studying the interface-stabilizing properties of materials with reduced solubility, such as certain plant protein extracts. The presence of such particles should be carefully considered when conducting pendant drop tensiometry.

## 1 Introduction

Multiphase systems are ubiquitous in daily life, and common examples are emulsions (oil–water or water–oil) or foams (air–water). These systems contain surface-active molecules, such as low molecular-weight surfactants and biopolymers (e.g., protein), to stabilize the interface.^1−4^ The effectiveness of these molecules to form and stabilize the interface can be evaluated by studying their interfacial properties.^5−7^ Various techniques are available to assess the interfacial properties, of which drop tensiometry is the most commonly used.^8,9^ Drop tensiometry relies on image analysis for determining the shape of a droplet, which is subsequently fitted with the Young–Laplace equation^10,11^ to determine the surface tension. Therefore, there are some limitations to its applicability. In a rising drop mode (also known as the bubble method), for example, the turbidity of the aqueous (bulk) phase surrounding the droplet reduces the sharpness of the image. Therefore, the pendant (or hanging) drop method is more suitable for the analysis of turbid samples, as the outer (bulk) phase either consists of air or (stripped/clear) oil, resulting in a sharp image of the droplet.

For this reason, turbid (and often less soluble) samples, such as phospholipids/lecithin, (aggregated) proteins, or antifoamers,^12−20^ are generally analyzed using the pendant drop configuration. Particles causing turbidity may, however, sediment in time. Even though multiple studies analyzed samples with insoluble materials, the impact of possible sedimentation on the interfacial properties has not been evaluated yet.

In drop tensiometry, the surface tension is determined by fitting the droplet shape with the Young–Laplace equation (eq 1).^21^
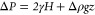
1Here, Δ*P* = *P*_in_ – *P*_out_; *P*_in_ is the pressure in the interior of the droplet, *P*_out_ is the pressure in the outer phase, γ is the surface tension, and *H* is the curvature of the interface, Δρ = ρ_in_ – ρ_out_; ρ_in_ is the droplet density, ρ_out_ is the continuous phase density, *g* is the gravitational acceleration [9.81 m/s^2^], and *z* is the coordinate measured along the vertical axis of the droplet. According to eq 1, the curvature of the droplet plays a crucial role in surface tension calculation if we assume a constant density difference (between the droplet and the continuous phase).

The shape (and curvature) of the droplet depends on the interplay between gravitational and interfacial forces, and both contributions are present in the Young–Laplace equation (eq 1).

The surface tension causes the formation of spherical droplets/bubbles, while gravity (vertically) elongates them.^8,11^ Adsorption of surface-active components decreases surface tension, leading to a more dominating contribution of gravity and thereby a more elongated droplet. One can argue that sedimentation, leading to the accumulation of particles at the apex of the droplet, may also impact the droplets’ shape. Since any changes in the droplet shape will affect the surface tension, sedimentation potentially influences the value of the measured surface tension, which could erroneously be attributed to adsorption/desorption processes of the surface-active components.

In this study, we will determine the impact of sedimenting particles in tensiometry experiments on an air–water interface using a model system consisting of non surface-active glass beads and whey protein isolate (WPI). Whey protein was chosen, as interfacial films formed by this protein are well-characterized.^22−25^ We have chosen inert non-food grade glass particles in this study to decouple the effects of sedimentation and adsorption and exclude interactions with the proteins. In addition to adsorption behavior, the rheological properties of the air–water interface were investigated by applying large amplitude oscillatory dilatational deformations on the whey protein-stabilized interface in the absence and presence of sedimenting glass beads. The results yield crucial insights on the impact of an often-neglected factor in the analysis of interfacial properties, allowing improved design and more accurate execution of interfacial analyses.

## 2 Results and Discussion

### 2.1 Characterization of Glass Bead Dispersions

**Figure 1:**
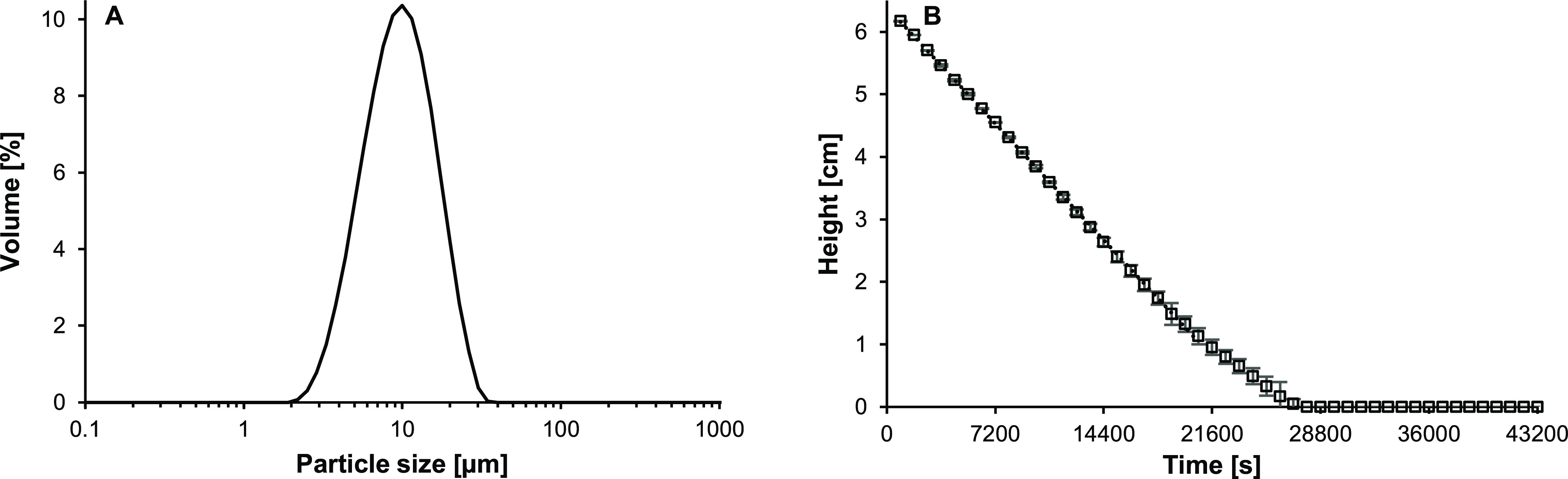
(A) PSD of glass beads and (B) height of the glass bead layer in time. Averages and error bars in panel B were obtained from duplicate measurements.

The glass beads have a wide particle size distribution (PSD) ranging from 2.2 to 34.7 μm with a *d*_3,2_ of 7.7 ± 0.2 μm (Figure 1A). This wide size distribution is in line with previous observations made by scanning electron microscopy.^28^ Due to their relatively large particle size, the glass beads sedimented rapidly, with an experimental sedimentation velocity of 2.59 × 10^–4^ cm/s (= 0.93 cm/h; Figure 1B; determined by Turbiscan). Based on this sedimentation velocity and Stokes’ law (eq 2), the theoretical particle diameter was 1.77 μm. This is in close agreement with the smallest particles determined by static light scattering (Figure 1A), which was expected, as the height of the glass bead layer is dictated by the sedimentation of the smallest particle able to reduce the transmission of light.

From this, the amount of sedimented particles over time was determined. It was estimated based on the PSD (Figure S1D in the Supporting Information). It was found that the majority of the glass beads (i.e., 99.99%) had sedimented within 3 h.

### 2.2 Interfacial Adsorption Behavior

#### 2.2.1 Impact of Glass Beads on the Surface Pressure of Pure Water Interfaces

In the rising droplet (or bubble) method, the surface tension of a 0.2% (w/w) glass bead dispersion was constant at 71.1 ± 0.7 mN/m for 12 h (Figure S3 in the Supporting Information). This value is close to the surface tension of ultrapure Milli-Q water (i.e., 71.3 ± 0.6 mN/m), indicating that the glass beads do not adsorb to the air–water interface.

**Figure 2:**
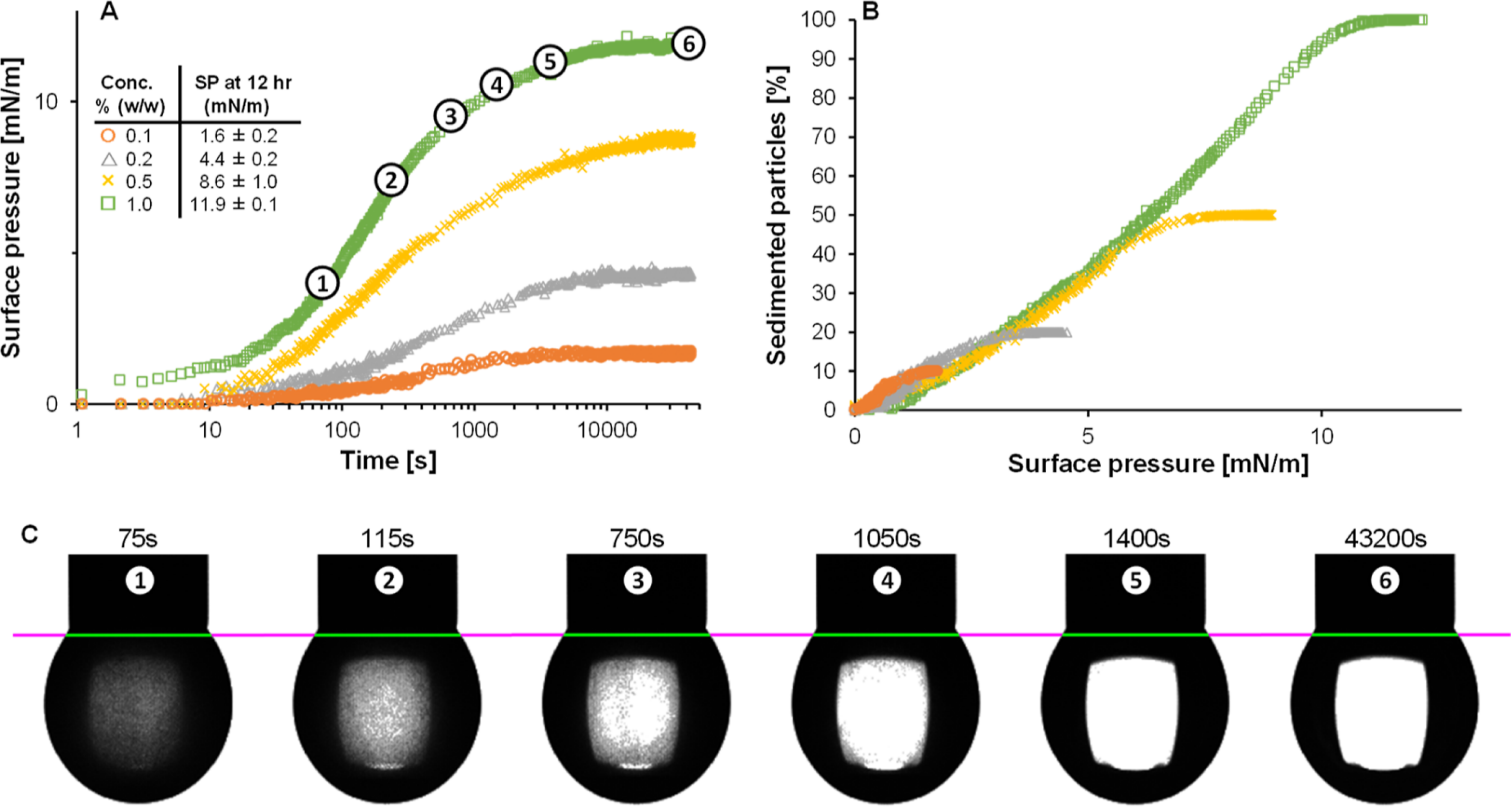
Surface pressure as a function of time (A) and the relative amount of sedimented particles as a function of surface pressure (B) for 0.1% (brown ring open), 0.2% (ash triangle up open), 0.5% (yellow multiplication), and 1% (green box) (w/w) glass beads. Screenshots of the pendant droplet containing 1% (w/w) glass beads at various time points (C). Inset in panel A shows the surface pressure at various concentrations after 12 h of adsorption. The numbers in panel C correspond to the numbers in panel A. The purple line is a line for the image analysis. The surface pressure curves are averages obtained from at least duplicate measurements, and the amount of sedimented particles is expressed relative to 1% (w/w) glass beads.

In the pendant drop method, the glass bead dispersions significantly lowered the surface tension at all concentrations, as shown by the surface pressure increase (Figure 2A). The surface pressure increase clearly levels off after 3 h, corresponding to the time at which nearly all glass beads sedimented (Figures 2C and S1D in the Supporting Information). In addition, a clear concentration dependence of the surface pressure increase was observed. At the lowest concentration of 0.1% (w/w), the surface pressure increased to 1.6 ± 0.2 mN/m after 12 h, which increased up to 11.9 ± 0.1 mN/m for a 1.0% (w/w) glass bead dispersion. Since the glass beads were shown to be non surface-active, this surface pressure increase is postulated to be caused by sedimentation, rather than adsorption, of the glass beads.

One might argue that sedimentation could force the glass beads onto the interface. To verify this possible explanation, the rheological properties of the droplet’s interface were studied. The rheological response of the droplet with glass beads was similar to that of a water droplet (data not shown), suggesting no absorption of the glass beads. Alternatively, sedimentation leads to sediment formation at the droplet apex, resulting in an increase in the gravitational forces on the droplet. Therefore, the droplet curvature is altered, which is erroneously analyzed by the software as an increase in (apparent) surface pressure. To verify this hypothesis, the amount of sedimented particles was plotted as a function of (apparent) surface pressure (Figure 2B). This results in a single master curve for the different concentrations, in which the (apparent) surface pressure linearly increases with an increasing amount of sedimented particles (i.e., increasing gravitational force). The linear increase with increasing gravitational force underlines the hypothesis that sedimentation leads to an (apparent) surface pressure increase.

The linear relationship between sedimented material and apparent surface pressure can be explained using the Young–Laplace equation (eq 1). The solution of this equation is influenced by an interplay between interfacial forces (after surfactant adsorption) and gravitational forces. In view of the low concentration of glass beads, the overall density of the droplet is close to the density of water in the initial adsorption phase (<10 s), as the surface tension was close to 71 mN/m. The sedimentation of particles onto the droplet apex leads to a local increase in density. This change in droplet density is not corrected during the analysis, which contributes to the increase in the apparent surface pressure, as shown in Figure 2A. Since eq 1 is linear in Δρ, a linear increase in surface pressure is shown in Figure 2B. In addition, we created a water droplet in the air and increased the droplet density in the analysis settings (Figure S2 in the Supporting Information). Here, we observe a linear increase in (apparent) surface tension at a higher set droplet density, which further underpins the curve in Figure 2B, as we also found a near-linear relationship between surface pressure and the amount of sedimented particles (i.e., higher droplet density).

#### 2.2.2 Impact of Glass Beads on the Surface Pressure of Whey Protein-Stabilized Interfaces

**Figure 3:**
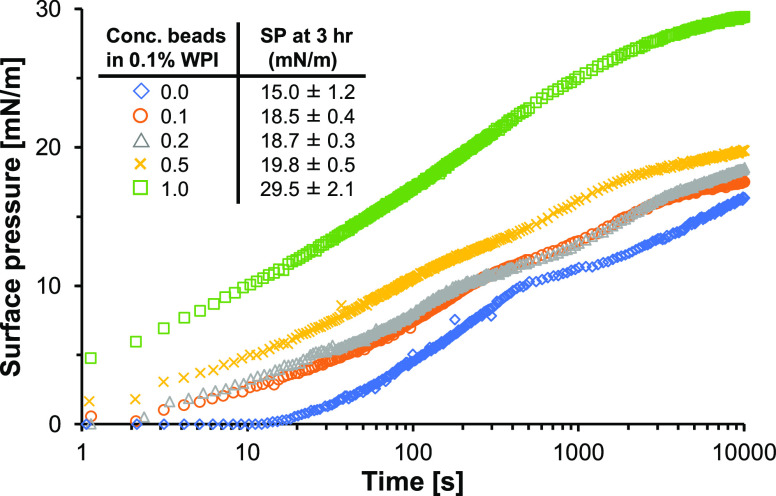
Surface pressure as a function of time of 0.1% (w/w) WPI with 0.0% (blue tilted square open), 0.1% (brown ring open), 0.2% (ash triangle up open), 0.5% (yellow multiplication), and 1% (green box) (w/w) glass beads. The surface pressure curves are averages obtained from at least duplicate measurements.

Sedimenting material might also affect the properties of a protein-stabilized interface. This effect was studied by mixing WPI with glass beads (Figure 3). For pure WPI, a lag phase of roughly 10 s was observed. Apparently, insufficient material was adsorbed at the interface during this initial phase.^29^ In time, more proteins are adsorbed, thereby increasing the surface pressure to 15.0 ± 1.2 mN/m after 3 h of adsorption.

Surprisingly, the addition of as little as 0.1% (w/w) glass beads resulted in a rapid increase of the (apparent) surface pressure in the initial phase (Figure 3). This was unexpected, as the pure 0.1 (w/w) glass bead dispersion as well as the pure 0.1% (w/w) WPI solution showed a substantially slower increase in surface pressure (Figure 2A). This discrepancy was postulated to be caused by a difference in ionic strength. Indeed, a 0.1% (w/w) WPI solution with corrected conductivity (i.e., 0.57 mM NaCl, equivalent to a 1% glass bead dispersion) also rapidly increased the surface pressure in the initial phase (Figure S4 in the Supporting Information). As a result, the rapid increase in (apparent) surface pressure for the mixtures of WPI with 0.1 and 0.2% (w/w) glass beads was primarily attributed to the increased ionic strength on the adsorption of WPI rather than sedimentation. The increased ionic strength screens the protein charge, reducing the electrostatic repulsion between the protein molecules and between proteins and the interface. As a consequence, the electrostatic barrier for adsorption also decreases, leading to more rapid adsorption.^30^ It should be noted that the value of the surface pressure after 3 h was not affected by the increase in ionic strength. Also, the mechanical properties of the interface were not affected by higher ionic strength, as the Lissajous plots of WPI with and without added salt were similar (Figure S5 in the Supporting Information).

Increasing the glass bead concentration leads to a higher apparent surface pressure (Figure 3), as was also observed for the pure glass beads (Figure 2A). In the mixture with 1% (w/w) glass bead, the apparent surface pressure even increased from ±14.5 to 29.5 mN/m after 3 h of adsorption. Consequently, sedimenting material clearly also affects the droplet shape with adsorbed proteins, leading to higher apparent surface pressure. These findings indicate a significant impact of sedimenting material in the pendant droplet method, especially with respect to surface tension/pressure analysis.

### 2.3 Surface Dilatational Rheology

#### 2.3.1 Impact of Glass Beads on the Surface Dilatational Moduli of Whey Protein-Stabilized Interfaces

**Figure 4:**
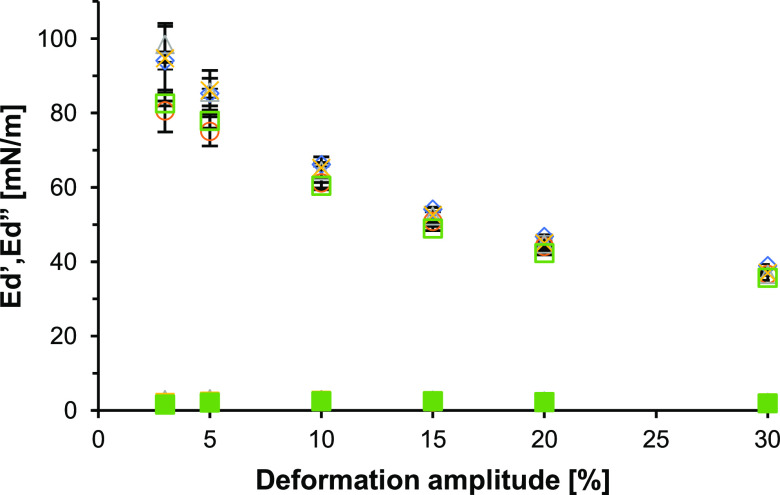
Surface dilatational moduli as a function of deformation amplitude of 0.1% WPI with 0.0% (blue tilted square open), 0.1% (brown ring open), 0.2% (ash triangle up open), 0.5% (yellow multiplication), and 1% (green box). The open symbols indicate the *E*_d_′, and the filled symbols indicate *E*_d_″. Averages and error bars were obtained from triplicate measurements.

Sedimentation might also affect the rheological properties of the interfaces. Therefore, amplitude sweeps were performed by increasing the oscillatory deformation amplitude from 3 to 30% to determine the surface dilatational moduli (Figure 4). In contrast to the glass bead-filled droplets, the whey protein-stabilized interfaces showed a markedly different rheological response with an elastic dilatational modulus (*E*_d_′) of 94.1 ± 3.4 mN/m at 3% deformations, decreasing to 38.9 ± 0.5 mN/m at 30% deformation. The viscous component of the modulus (*E*_d_″) had low values, varying from 0.5 to 1.4 mN/m over the whole range of deformations. This implies a substantially higher *E*_d_′ than the *E*_d_″, suggesting viscoelastic solid-like behavior. The decay of *E*_d_′ upon higher deformations suggests the disruption of the microstructure of the WPI-stabilized interfacial layer. Higher deformations can affect the mechanical properties of the WPI-stabilized interface, leading to lower stiffness of the interfacial layers. Whey proteins interact strongly at the air–water interface, allowing the formation of stiff interfacial layers, as shown in previous studies.^22,31,32^

The addition of 0.1–1% (w/w) glass beads to the system led to comparable *E*_d_′ varying from 82.6 to 98.4 mN/m at the lowest deformation of 3% and decreasing from 35.7 to 37.0 mN/m at 30% deformation. The moduli at lower deformations (3 and 5%) seem to deviate from those of a pure WPI-stabilized interface. However, the WPI-glass bead mixtures had high standard deviations of, for instance, 5.6–9.3 mN/m at 3% deformation. When considering these standard deviations, the moduli values in the lower deformations (3 and 5%) are not substantially different.

#### 2.3.2 Impact of Glass Beads on the Lissajous Plots of Whey Protein-Stabilized Interfaces

**Figure 5:**
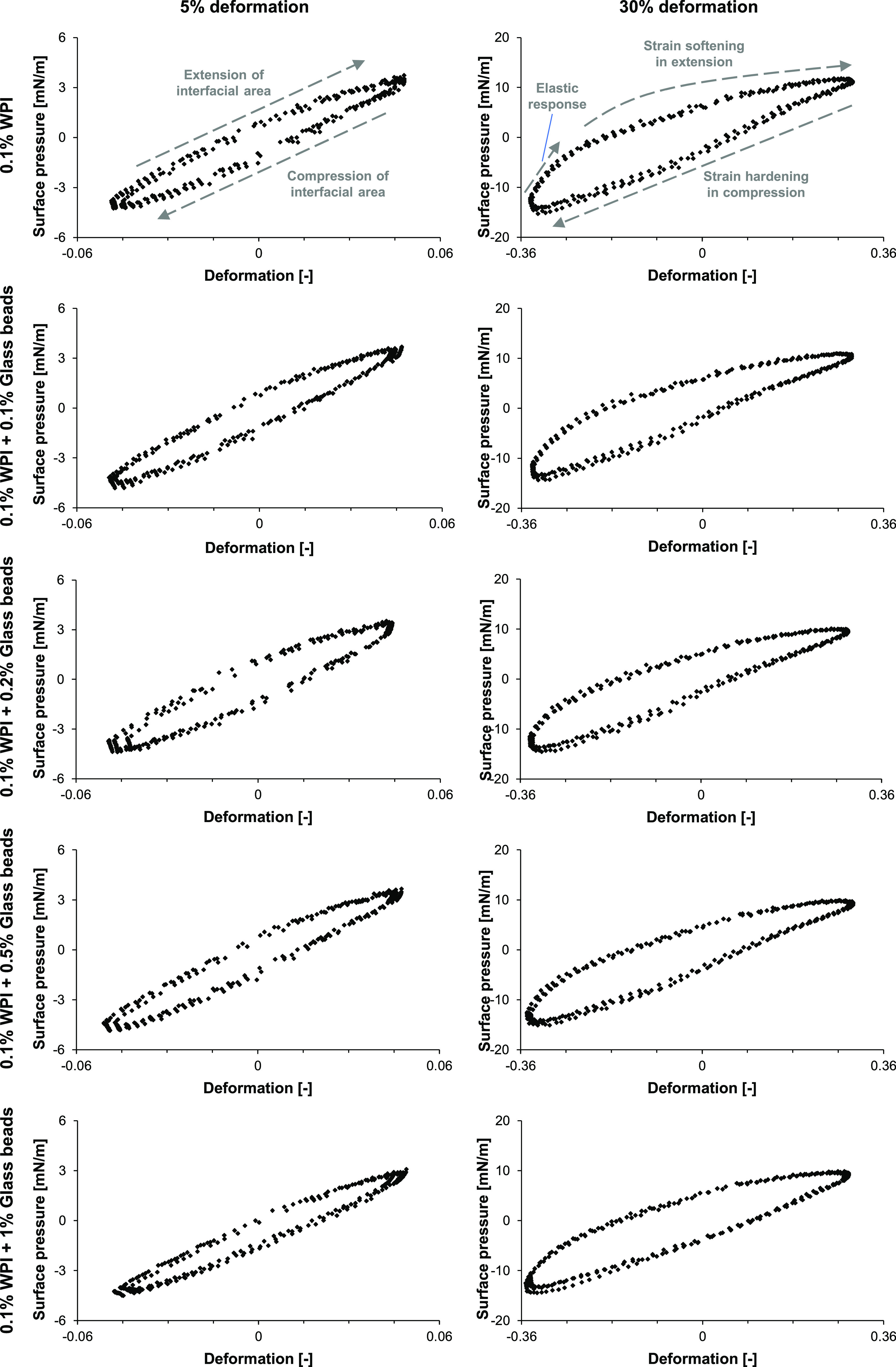
Lissajous plots of 0.1% WPI with 0, 0.1, 0.2, 0.5, and 1% glass beads for 5 and 30% deformation. A representative Lissajous plot is shown for each sample, and comparable plots were obtained from triplicate measurements.

A more accurate method to analyze nonlinear oscillatory deformations (in both dilatational and shear rheology) is by plotting Lissajous plots of the (surface) stress versus deformation. Lissajous plots supplement the surface dilatational moduli, as non-linearities of the rheological response are incorporated^10,33^ (Figure 5).

The Lissajous plots follow a clockwise movement, where the upper side of the plot is the extension part of the cycle of the interface, and the lower side is the compression part of the cycle. The shape of the plots reveals whether the elastic or viscous component of the rheological response is dominating. A fully elastic response is expressed as a closed plot (straight line, no phase shift of the stress response), while a fully viscous response is shown as a circle (90° phase shift of the stress response). In previous work, protein-stabilized interfaces showed viscoelastic responses, which are characterized by an ellipsoidal shape.^22,34,35^ The same ellipsoidal shape is present at 5% deformation for a WPI-stabilized air–water interfacial film (Figure 5).

At higher deformations, such as 30%, the Lissajous plot of WPI became wider, suggesting a relative increase in the viscous contribution to the stress response. Another development at higher deformations is the pronounced asymmetry between the extension and compression cycle of the plot. These asymmetries result from non-linearities in the stress response, which are usually neglected in moduli obtained from the first harmonic of the Fourier spectrum. We explain the asymmetries stepwise by starting in the bottom-left corner of the 30% deformation plot of WPI. This point is the start of the extension cycle, where we see a steep increase in surface pressure, indicating a (predominantly) elastic response. Around a deformation of −0.27, the slope of the curve decreases gradually until the end of the extension cycle. Here, the previously dominating elastic component gradually diminishes, and the viscous contribution to the surface pressure response increases. Two phenomena upon such large deformation occur: (1) the interfacial microstructure loses its cohesiveness, leading to a reduction in stiffness, and (2) the interfacial layer is stretched, diluting the adsorbed proteins. The overall behavior in this cycle is called strain softening in extension.

We observe the opposite in the compression cycle, as the surface pressure reaches higher (absolute) values (−16 mN/m) compared to the extension cycle (+12 mN/m). This behavior is known as strain hardening in compression, which was previously attributed to the jamming of densely clustered adsorbed proteins. The explained behavior for the 30% Lissajous plot of a WPI-stabilized interface suggests the presence of strong in-plane interactions among adsorbed proteins. This is in line with the previous work in which a WPI-stabilized interface was suggested to exhibit the rheological behavior of a viscoelastic solid.^22,36^

The Lissajous plots of WPI-glass bead mixtures were remarkably similar to the ones of pure WPI. A minor difference is present when increasing the glass bead concentration. The Lissajous plots of 0.1% WPI with 1% glass beads were slightly more tilted toward the horizontal axis. For instance, at 30% deformation, maximum values in compression changed from −16 to −14 mN/m, and those in extension decreased from +10 to +9 mN/m. Therefore, the presence of sedimented glass beads in a pendant drop seems to only marginally affect the dilatational rheological properties of a protein-stabilized interface by slightly reducing the stiffness of the protein-stabilized interfacial film.

### 2.4 Is the Impact of Glass Beads Dominated by an Absolute Amount or a Protein-Glass Bead Ratio?

**Figure 6:**
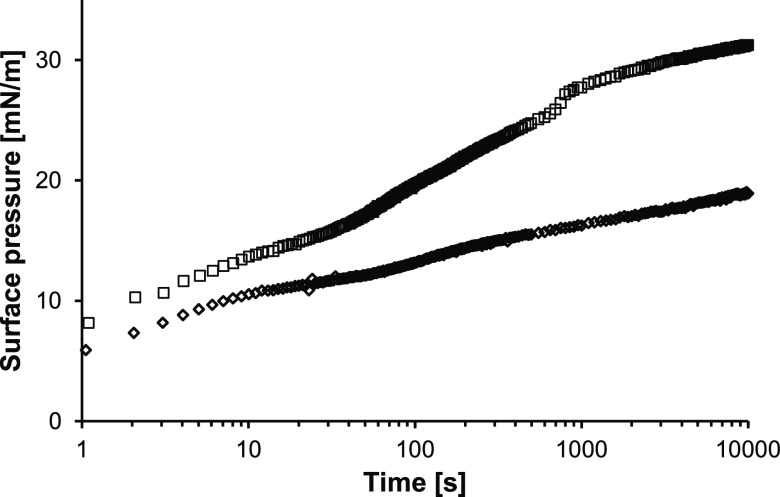
Surface pressure as a function of time of 1% (w/w) WPI with 0% (blue tilted square open) and 1% (blue box) glass beads. The surface pressure curves are averages obtained from at least duplicate measurements.

In previous sections, glass beads were shown to significantly affect the (apparent) surface pressure of a protein-stabilized interface. An important question when observing effects in mixtures is whether the absolute amount or the ratio between the components is dominant. Therefore, the interfacial properties of 1% (w/w) WPI with 1% (w/w) glass beads (i.e., a protein-to-glass bead ratio of 1:1) were determined (Figure 6).

The surface pressure of pure WPI increased rapidly from 5.9 mN/m at 1 s to 18.9 mN/m at 10,800 s. The presence of 1% glass beads led to vastly higher apparent surface pressures from 8.2 mN/m at 1 s to 31.4 mN/m at 10,800 s. This shows that the sedimenting glass beads also affected the droplet shape at a protein concentration of 1%, leading to a higher apparent surface pressure. This is in line with the observation for the 0.1% WPI solutions (Figure 3).

A clear difference can be observed when comparing the effect of the addition of glass beads on the apparent surface pressure for samples with different absolute amounts of glass beads (Table 1). While the apparent surface pressure increased by only ±2.5 mN/m when adding 0.1% glass beads to 0.1% WPI, it increased by a pronounced 12.5 mN/m when adding 1% glass beads to 1% WPI. This is in line with the clear increase in apparent surface pressure with an increasing amount of sedimented glass beads (Figure 2B). When comparing the effects of protein-to-glass bead ratios (i.e., 1:1 and 1:10) at a constant absolute amount of glass beads (i.e., 1%), the apparent surface pressure increase was similar (i.e., 14.5 and 12.5 mN/m for 0.1% WPI and 1% WPI solutions, respectively). Therefore, it was concluded that the absolute amount of sedimenting material is the dominant factor affecting the droplet shape and thus the apparent surface pressure.

**Table 1:** Overview of the Protein and Glass Bead Concentration, Protein-to-Glass Bead Ratio, and Surface Pressure after 3 h of Adsorption of 0.1% WPI Mixed with 0.1 and 1% Glass Beads and 1% WPI Mixed with 1% Glass Beads

*C*_protein_ [% (w/w)]	*C*_glass bead_ [% (w/w)]	protein-to-glass bead ratio [−]	surface pressure increase [mN/m]	reference
0.1	0.1	1:1	2.5	0.1% WPI
1	1	1:1	12.5	1% WPI
0.1	1	1:10	14.5	0.1% WPI

Finally, the rheological properties of the 1% WPI with and without 1% glass beads were studied (Figure S6 in the Supporting Information). Here, the impact of the glass beads on the rheological properties was minimal compared to the impact of sedimentation on the apparent surface pressure, as was also observed for 0.1% WPI (Figure 5).

## 3 Conclusions

The impact of sedimenting material on the analysis of the interfacial properties by pendant drop tensiometry was evaluated. Sedimentation of the (non surface-active) glass beads decreased the apparent surface tension of the pure air–water interface and the interface with adsorbed whey proteins in a similar manner. This decrease is postulated to be caused by an increase in local density at the apex of the droplet, leading to an alteration of the droplet shape. The shape alteration is erroneously analyzed using the software as a decrease in apparent surface tension because the change in droplet density is not corrected during analysis. The effect of sedimenting material on the (apparent) surface pressure is dictated by the absolute amount of glass beads rather than the protein-to-glass bead ratio. In contrast to the surface tension, sedimentation had only minor effects on the rheological properties, which showed only slightly weaker interactions among adsorbed proteins. This is most likely because the particles do not actually adsorb at the interface. They merely increase the shear viscosity of the bulk phase close to the interface, and during deformation, this causes only a slight increase in the viscous stress exerted by the bulk phase on the interface. However, this effect is negligible at the low frequencies used here.^37^

In short, non surface-active insoluble/sedimenting material can tremendously affect pendant drop tensiometry measurements by increasing the gravitational contribution. Its impact should be carefully examined to increase the accuracy of these analyses, especially for biopolymer systems with low (water) solubility, such as plant proteins and lecithins.

## 4 Experimental Section

### 4.1 Materials

Uncoated soda lime solid glass microspheres (glass beads; Product no: P2011SL, Cospheric, USA), BiPRO WPI (WPI; ±74% β-lactoglobulin;^26^ Davisco Foods International, USA), and all other chemicals (Sigma-Aldrich, USA) were used as received.

### 4.2 Sample Preparation

#### 4.2.1 Glass Beads

Glass beads were dispersed in Milli-Q water (Milli-Q Purelab Ultra, Germany) at concentrations varying from 0.1 to 2% (w/w). The glass bead dispersions were put on an overhead stirrer for 1 h. The samples were transferred into 50 mL centrifuge tubes. The sample was centrifuged at 1,000*g* for 10 min. The supernatant was carefully removed by pipetting. The remaining pellet was redispersed in Milli-Q water (to obtainan initial weight) and centrifuged again. This washing process was repeated once more. Washing of the glass bead dispersions was required to remove salts. Subsequently, the sample was high-speed sheared using a T25 Ultra Turrax (Ika, Germany) at 10,000 rpm for 30 s to break up any clusters.

#### 4.2.2 WPI

WPI was dissolved in Milli-Q water at concentrations varying from 0.1 to 2% (w/w). The sample was carefully stirred for 1 h and then stored at 4 °C to allow overnight hydration. On the following day, the pH of the WPI solution was adjusted to 7.0 using 0.1 or 1 M NaOH.

#### 4.2.3 Mixtures of WPI and Glass Beads

Mixtures of WPI and glass beads were prepared by preparing the WPI solution and glass bead solution separately at two times higher concentrations than the target concentration in the mixture. Subsequently, both solutions were mixed in a 1:1 (v/v) ratio. Afterward, the pH was adjusted to 7.0.

In this study, an essential aspect is to avoid interactions between the glass beads and WPI. Based on the supplier’s information, the glass beads were expected to have a hydrophilic surface, which is slightly negatively charged at neutral pH. The hydrophilic nature of the surface was confirmed in a previous study by determining the contact angle of the glass beads, which was 48°.^27^ As a result of their similar charge, whey proteins and glass beads were expected to repel each other.^22^

To check this, the potential interaction between proteins and glass beads was evaluated by creating a 0.1% (w/w) WPI solution with and without 1% (w/w) glass beads. Both solutions were centrifuged at 1,000*g* for 10 min to sediment the glass beads. The supernatant was collected by pipetting, and the protein content of the supernatant was determined using a Bradford assay. WPI solutions (also centrifuged) with concentrations ranging from 0.01 to 0.15% (w/w) were included to create a calibration curve. Aliquots of 50 μL of sample were mixed with 1.5 mL of Bradford reagent (Sigma-Aldrich, USA) in cuvettes. The samples were equilibrated in the dark and at room temperature for 5 min, followed by absorbance measurement at a wavelength of 595 nm using a spectrophotometer. All samples were prepared in triplicate and measured in duplicate. The outcome was 0.100 ± 0.002 and 0.099 ± 0.002% (w/w) for the WPI solution without and with glass beads, respectively, after centrifugation. Therefore, the protein concentration of the continuous phase of the WPI-glass bead solution (after removal of glass beads) and the pure WPI sample was not significantly different, suggesting that the adsorption of WPI on the glass beads is negligible.

### 4.3 Particle Size Analysis

The particle size of the glass beads was analyzed by static light scattering in a Mastersizer 2000 (Malvern Instruments, UK). The refractive indices used were 1.50 for the glass beads (according to the manufacturer) and 1.33 for the water phase. The measurements were performed in triplicate.

### 4.4 Sedimentation Analysis

The sedimentation velocity of the particles was determined based on the height of the particle layer in time and analyzed by static light scattering in a Turbiscan MA 2000 (Formulaction, France). The transmittance of 1% (w/w) glass bead dispersions was acquired at room temperature for 12 h with intervals of 15 min. The measurements were performed in duplicate. Subsequently, the height of the particle layer was determined as the height at which the transmittance dropped below 0.99. For clarity, the height of the particle layer is dominated by the smallest particles in the system, as larger particles sediment more rapidly. This analysis was performed to prove that glass beads sediment in time. Based on linear regression of the decrease of the particle layer height in time, the sedimentation velocity was obtained. From the experimental sedimentation velocity, the theoretical particle diameter was estimated using Stokes’ law (eq 2).
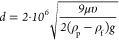
2

In which *d* is the particle diameter [μm], μ is the viscosity of the continuous phase [1.0016 × 10^–3^ kg/(m s)], υ is the experimental sedimentation velocity [m/s], ρ_p_ is the particle density [that is 2520 kg/m^3^],^27^ and ρ_f_ is the density of the continuous phase [998.23 kg/m^3^ at 20 °C].

### 4.5 Calculation of the Theoretical Amount of Sedimented Particles in Time

The theoretical amount of sedimented particles in time was estimated from the PSD determined by the Mastersizer. First, the cumulative PSD (*P* [%]) was determined from the experimentally obtained PSD (Section 2.3) data using eq 3.
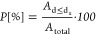
3

In which *A*_d≥dx_ is the area under the PSD curve for particles with a diameter ≤ diameter *x*, and *A*_total_ is the total area under the PSD curve.

Then, for each particle size, the theoretical sedimentation velocity was calculated using eq 2, and the cumulative PSD was converted into the sedimentation velocity distribution (Figure S1B in the Supporting Information). To calculate the number of particles sedimented at time *t*, we need to account for the vertical position of the particles in the tubing, needle, and droplet, as particles will have a different sedimentation distance in each location. Assuming a homogeneous distribution of particles at the start of the measurement, the total height of the system (i.e., 4.5 cm) was divided into 100 equidistant sedimentation heights with steps of 0.045 cm. For each sedimentation height, the amount of sedimented particles in time was calculated based on the sedimentation distance and the sedimentation velocity distribution. The amount of sedimented particles in time was fitted with a lognormal distribution using the least squares method (Figure S1C in the Supporting Information). Based on the fitting parameters, the theoretical amount of sedimented particles in time could be determined for each sedimentation height. Finally, all sedimentation heights were averaged over the PSD, and the volume was assumed to be identical for each sedimentation height bin (Figure S1D in the Supporting Information).

### 4.6 Determination of Interfacial Properties

#### 4.6.1 Rising Drop Method

The rising drop method was used to determine the potential surface activity of glass beads using an automated drop tensiometer (ADT, Teclis Scientific, France). A glass cuvette was filled with glass bead dispersion (0.2% w/w), WPI solution (0.1% w/w), or a WPI-glass bead mixture (0.1% WPI + 0.2% glass beads). A rising air bubble with an area of 20 mm^2^ was formed at the tip of a curved G18 needle. A camera monitored the air bubble shape, which was transformed into surface tension values by fitting with the Young–Laplace equation (eq 1). The surface tension was measured for 12 h. Samples containing glass beads were stirred with a magnetic stirring rod in the cuvette until the start of the analysis. The measurements were performed at least in duplicate.

#### 4.6.2 Pendant (or Hanging) Drop Method

The impact of sedimenting glass beads on interfacial properties was studied by the pendant drop method in a PAT-1M drop tensiometer (SINTERFACE Technologies, Germany). A pendant (or hanging) drop with a 20 mm^2^ surface area was created at the tip of a needle. The surface tension was determined similarly as mentioned in paragraph 2.6.1. First, a droplet containing glass bead dispersion (0.1–1.0% w/w), protein solution (0.1 or 1.0% w/w), or a mixture (0.1–1.0% WPI with 0.1–1.0% glass beads) was created, and the area was kept constant for 12 h to obtain surface pressure isotherms. Since sedimentation of the glass beads may affect the quality of the droplet shape analysis, the deviation of the droplet shape from a Laplacian shape was evaluated using the drop tensiometer software. The deviation for a 1% (w/w) glass bead dispersion varied between 0.10 and 0.16% when increasing the surface pressure to 11.9 mN/m. In comparison, the deviation for a 0.1% (w/w) WPI solution in the same surface pressure range was 0.10–0.12%. These values are well within the manufacturers’ specification for a good fit with the Laplacian model (i.e., deviation <1%). As a result, droplets with sedimented glass beads can be accurately analyzed using the Young–Laplace equation.

The surface pressure (Π) was calculated using eq 4.
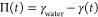
4Here, the γ_water_ is the surface tension of the pure water–air interface, and γ(*t*) is the surface tension of the interface at time *t*.

Amplitude sweeps were performed after 3 h of waiting time since nearly all glass beads (i.e., 99.99%) sedimented within this timespan (Figure S1D in the Supporting Information). The oscillatory deformations were performed by increasing the deformation amplitude from 3 to 30% at a fixed oscillatory frequency of 0.02 Hz. For each deformation amplitude, five oscillations were performed, followed by a 50 s pause. The measurements were performed at least in duplicate. Lissajous plots were constructed from the middle three oscillations by plotting the surface stress (γ – γ_0_) against the deformation ((*A* −*A*_0_)/*A*_0_). Here, γ and *A* are the surface tension and area of the deformed interface, and γ_0_ and *A*_0_ are the surface tension and area of the non-deformed interface.

## Supporting Information Available

PSD of the glass beads and supporting graphs on the calculation of the theoretical sedimented amount of particles;surface tension as a function of set droplet phase density in the tensiometer; surface tension as a function of time of glass beads, measured using a rising drop method; surface tension as a function of time of WPI with and without the presence of NaCl (conductivity equal to 1% glass beads); Lissajous plots of WPI with and without the presence of NaCl (conductivity equal to 1% glass beads); and Lissajous plots of 1% WPI with and without 1% glass beads (PDF)

## References

[ref1] Rodríguez Patino J. M.; Carrera Sánchez C.; Rodríguez Niño M. R. Implications of Interfacial Characteristics of Food Foaming Agents in Foam Formulations. Adv. Colloid Interface Sci. 2008, 140, 95–113. 10.1016/j.cis.2007.12.007. 18281008

[ref2] Murray B. S. Rheological Properties of Protein Films. Curr. Opin. Colloid Interface Sci. 2011, 16, 27–35. 10.1016/j.cocis.2010.06.005.

[ref3] Berton-Carabin C. C.; Sagis L.; Schroën K. Formation, Structure, and Functionality of Interfacial Layers in Food Emulsions. Annu. Rev. Food Sci. Technol. 2018, 9, 551–587. 10.1146/annurev-food-030117-012405. 29350560

[ref4] Delahaije R. J. B. M.; Gruppen H.; Giuseppin M. L. F.; Wierenga P. A. Toward Predicting the Stability of Protein-Stabilized Emulsions. Adv. Colloid Interface Sci. 2015, 219, 1–9. 10.1016/j.cis.2015.01.008. 25704489

[ref5] Sagis L. M. C.; Scholten E. Complex Interfaces in Food: Structure and Mechanical Properties. Trends Food Sci. Technol. 2014, 37, 59–71. 10.1016/j.tifs.2014.02.009.

[ref6] Dickinson E. Adsorbed Protein Layers at Fluid Interfaces: Interactions, Structure and Surface Rheology. Colloids Surf., B 1999, 15, 161–176. 10.1016/S0927-7765(99)00042-9.

[ref7] Wilde P.; Mackie A.; Husband F.; Gunning P.; Morris V. Proteins and Emulsifiers at Liquid Interfaces. Adv. Colloid Interface Sci. 2004, 108–109, 63–71. 10.1016/j.cis.2003.10.011. 15072929

[ref8] Ravera F.; Loglio G.; Kovalchuk V. I. Interfacial Dilational Rheology by Oscillating Bubble/Drop Methods. Curr. Opin. Colloid Interface Sci. 2010, 15, 217–228. 10.1016/j.cocis.2010.04.001.

[ref9] Jaensson N.; Vermant J. Tensiometry and Rheology of Complex Interfaces. Curr. Opin. Colloid Interface Sci. 2018, 37, 136–150. 10.1016/j.cocis.2018.09.005.

[ref10] Sagis L. M. C.; Humblet-Hua K. N. P.; van Kempen S. E. H. J. Nonlinear Stress Deformation Behavior of Interfaces Stabilized by Food-Based Ingredients. J. Phys.: Condens. Matter 2014, 26, 464105 10.1088/0953-8984/26/46/464105. 25347358

[ref11] Ferri J. K.; Fernandes P. A. L.; McRuiz J. T.; Gambinossi F. Elastic Nanomembrane Metrology at Fluid-Fluid Interfaces Using Axisymmetric Drop Shape Analysis with Anisotropic Surface Tensions: Deviations from Young-Laplace Equation. Soft Matter 2012, 8, 10352–10359. 10.1039/C2SM26604K.

[ref12] Barac M. B.; Pesic M. B.; Stanojevic S. P.; Kostic A. Z.; Cabrilo S. B. Techno-functional properties of pea (Pisum sativum) protein isolates: A review. Acta Period. Technol. 2015, 46, 1–18. 10.2298/APT1546001B.

[ref13] Martin A. H.; Castellani O.; de Jong G. A. H.; Bovetto L.; Schmitt C. Comparison of the Functional Properties of RuBisCO Protein Isolate Extracted from Sugar Beet Leaves with Commercial Whey Protein and Soy Protein Isolates. J. Sci. Food Agric. 2019, 99, 1568–1576. 10.1002/jsfa.9335. 30144065

[ref14] Renkema J. M. S.; Lakemond C. M. M.; de Jongh H. H. J.; Gruppen H.; van Vliet T. The Effect of PH on Heat Denaturation and Gel Forming Properties of Soy Proteins. J. Biotechnol. 2000, 79, 223–230. 10.1016/S0168-1656(00)00239-X. 10867183

[ref15] Pham L. B.; Wang B.; Zisu B.; Adhikari B. Complexation between Flaxseed Protein Isolate and Phenolic Compounds: Effects on Interfacial, Emulsifying and Antioxidant Properties of Emulsions. Food Hydrocolloids 2019, 94, 20–29. 10.1016/j.foodhyd.2019.03.007.

[ref16] Shevkani K.; Singh N.; Kaur A.; Rana J. C. Structural and Functional Characterization of Kidney Bean and Field Pea Protein Isolates: A Comparative Study. Food Hydrocolloids 2015, 43, 679–689. 10.1016/j.foodhyd.2014.07.024.

[ref17] Pichot R.; Watson R. L.; Norton I. T. Phospholipids at the Interface: Current Trends and Challenges. Int. J. Mol. Sci. 2013, 14, 11767–11794. 10.3390/ijms140611767. 23736688 PMC3709755

[ref18] Yang J.; Waardenburg L. C.; Berton-Carabin C. C.; Nikiforidis C. V.; van der Linden E.; Sagis M. C. Air-Water Interfacial Behavior of Whey Protein and Rapeseed Oleosome Mixtures. J. Colloid Interface Sci. 2021, 602, 207–221. 10.1016/j.jcis.2021.05.172. 34119758

[ref19] Li J.; Wang X.; Zhang T.; Wang C.; Huang Z.; Luo X.; Deng Y. A Review on Phospholipids and Their Main Applications in Drug Delivery Systems. Asian J. Pharm. Sci. 2015, 10, 81–98. 10.1016/j.ajps.2014.09.004.

[ref20] Joshi K. S.; Jeelani S. A. K.; Blickenstorfer C.; Naegeli I.; Windhab E. J. Influence of Fatty Alcohol Antifoam Suspensions on Foam Stability. Colloids Surf., A 2005, 263, 239–249. 10.1016/j.colsurfa.2005.01.004.

[ref21] Berry J. D.; Neeson M. J.; Dagastine R. R.; Chan D. Y. C.; Tabor R. F. Measurement of Surface and Interfacial Tension Using Pendant Drop Tensiometry. J. Colloid Interface Sci. 2015, 454, 226–237. 10.1016/j.jcis.2015.05.012. 26037272

[ref22] Yang J.; Thielen I.; Berton-Carabin C. C.; van der Linden E.; Sagis L. M. C. Nonlinear Interfacial Rheology and Atomic Force Microscopy of Air-Water Interfaces Stabilized by Whey Protein Beads and Their Constituents. Food Hydrocolloids 2020, 101, 105466 10.1016/j.foodhyd.2019.105466.

[ref23] Davis J. P.; Doucet D.; Foegeding E. A. Foaming and interfacial properties of hydrolyzed β-lactoglobulin. J. Colloid Interface Sci. 2005, 288, 412–422. 10.1016/j.jcis.2005.03.002. 15927608

[ref24] Zhang X.; Zhang S.; Xie F.; Han L.; Li L.; Jiang L.; Qi B.; Li Y. Soy/whey protein isolates: interfacial properties and effects on the stability of oil-in-water emulsions. J. Sci. Food Agric. 2021, 101, 262–271. 10.1002/jsfa.10638. 32627183

[ref25] Hinderink E. B. A.; de Ruiter J.; de Leeuw J.; Schroën K.; Sagis L. M. C.; Berton-Carabin C. C. Early Film Formation in Protein-Stabilized Emulsions: Insights from a Microfluidic Approach. Food Hydrocolloids 2021, 118, 106785 10.1016/j.foodhyd.2021.106785.

[ref26] Butré C. I.; Wierenga P. a.; Gruppen H. Effects of Ionic Strength on the Enzymatic Hydrolysis of Diluted and Concentrated Whey Protein Isolate. J. Agric. Food Chem. 2012, 60, 5644–5651. 10.1021/jf301409n. 22583537

[ref27] Tsabet È.; Fradette L. Effect of the Properties of Oil, Particles, and Water on the Production of Pickering Emulsions. Chem. Eng. Res. Des. 2015, 97, 9–17. 10.1016/j.cherd.2015.02.016.

[ref28] Gravelle A. J.; Marangoni A. G.; Barbut S. Modulating Water Mobility in Comminuted Meat Protein Gels Using Model Hydrophilic Filler Particles. Lebensm.-Wiss. Technol. 2020, 129, 109376 10.1016/j.lwt.2020.109376.

[ref29] Beverung C. J.; Radke C. J.; Blanch H. W. Protein Adsorption at the Oil/Water Interface: Characterization of Adsorption Kinetics by Dynamic Interfacial Tension Measurements. Biophys. Chem. 1999, 81, 59–80. 10.1016/S0301-4622(99)00082-4. 10520251

[ref30] Foegeding E. A.; Luck P. J.; Davis J. P. Factors Determining the Physical Properties of Protein Foams. Food Hydrocolloids 2006, 20, 284–292. 10.1016/j.foodhyd.2005.03.014.

[ref31] Yang J.; Lamochi Roozalipour S. P.; Berton-Carabin C. C.; Nikiforidis C. V.; van der Linden E.; Sagis L. M. C. Air-Water Interfacial and Foaming Properties of Whey Protein - Sinapic Acid Mixtures. Food Hydrocolloids 2021, 112, 106467 10.1016/j.foodhyd.2020.106467.

[ref32] Rühs P. A.; Scheuble N.; Windhab E. J.; Fischer P. Protein Adsorption and Interfacial Rheology Interfering in Dilatational Experiment. Eur. Phys. J. Spec. Top. 2013, 222, 47–60. 10.1140/epjst/e2013-01825-0.

[ref33] Ewoldt R. H.; Hosoi A. E.; McKinley G. H. New Measures for Characterizing Nonlinear Viscoelasticity in Large Amplitude Oscillatory Shear. J. Rheol. 2008, 52, 1427–1458. 10.1122/1.2970095.

[ref34] Böttcher S.; Keppler J. K.; Drusch S. Mixtures of Quillaja Saponin and Beta-Lactoglobulin at the Oil/Water-Interface: Adsorption, Interfacial Rheology and Emulsion Properties. Colloids Surf., A 2017, 518, 46–56. 10.1016/j.colsurfa.2016.12.041.

[ref35] Zhan F.; Li J.; Shi M.; Wu D.; Li B. Foaming Properties and Linear and Nonlinear Surface Dilatational Rheology of Sodium Caseinate, Tannin Acid, and Octenyl Succinate Starch Ternary Complex. J. Agric. Food Chem. 2019, 67, 2340–2349. 10.1021/acs.jafc.8b06356. 30640476

[ref36] Rühs P. A.; Affolter C.; Windhab E. J.; Fischer P. Shear and and nonlinear subphase controlled interfacial rheology of β-lactoglobulin fibrils and their derivatives. J. Rheol. 2013, 57, 1003–1022. 10.1122/1.4802051.

[ref37] Sagis L. M. C. Dynamic Properties of Interfaces in Soft Matter: Experiments and Theory. Rev. Mod. Phys. 2011, 83, 1367–1403. 10.1103/RevModPhys.83.1367.

