# Supplementary material for: Impact of Particle Sedimentation in Pendant Drop Tensiometry
Source: Langmuir. 2022 Aug 9;38(33):10183–91. doi: 10.1021/acs.langmuir.2c01193 (PMC9404539; doi:10.1021/acs.langmuir.2c01193)
Supplement: Supplementary file 1 — la2c01193_si_001.pdf [file la2c01193_si_001.pdf]

Impact of particle sedimentation in pendant drop tensiometry

Roy Delahaije[a,c], Leonard M. C. Sagis[a*], Jack Yang[a,b]

a. Laboratory of Physics and Physical Chemistry of Foods, Wageningen University, Bornse Weilanden 9, 6708WG Wageningen, The Netherlands
b. Laboratory of Biobased Chemistry and Technology, Wageningen University, Bornse Weilanden 9, 6708WG Wageningen, The Netherlands
c. FrieslandCampina Innovation Centre, Bronland 20, 6708 WH Wageningen, The Netherlands

*Email: Leonard M.C. Sagis (leonard.sagis@wur.nl)

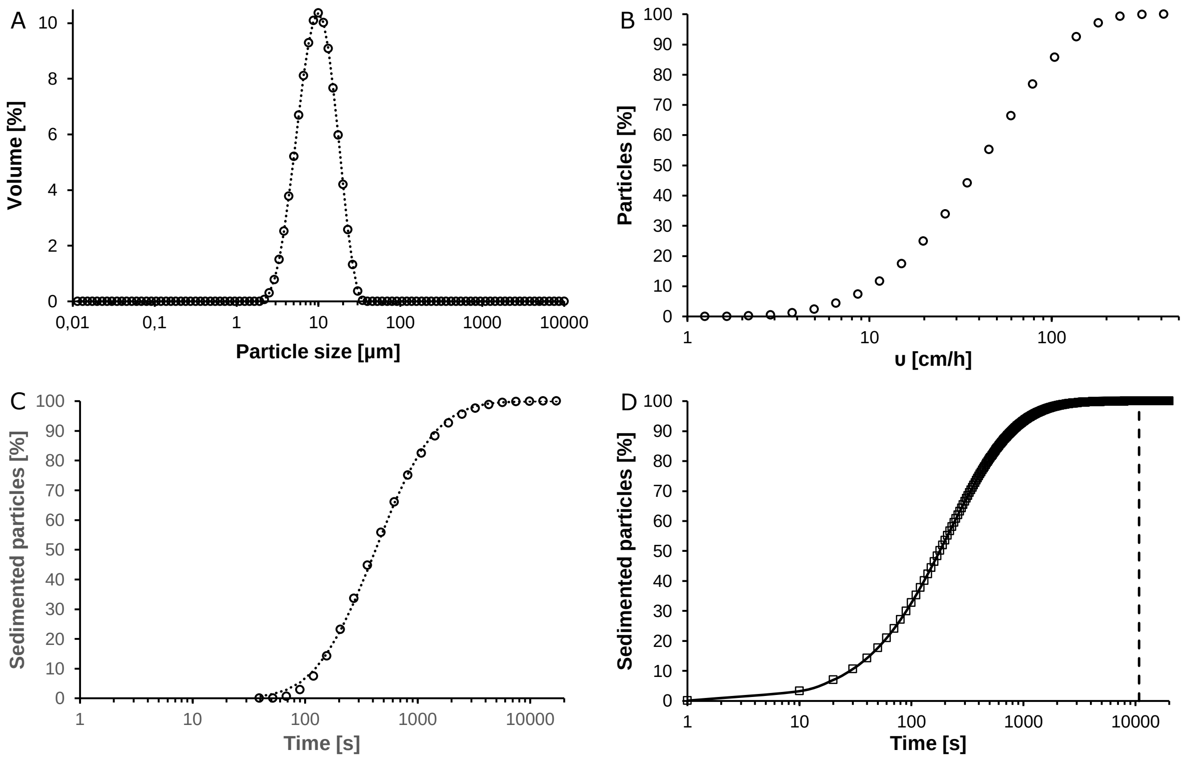

Figure S1. (A) The particle size distribution, (B) cumulative sedimentation velocity distribution calculated from the particle size distribution using equation 3 and 4, (C) amount of sedimented particles in time calculated from the cumulative sedimentation velocity distribution and sedimentation height of 4.5 cm, and (D) the amount of sedimented particles in time averaged over all sedimentation heights. The dotted line in panel C represents the lognormal distribution fit. The dashed line in panel D represents t = 10,800 s.

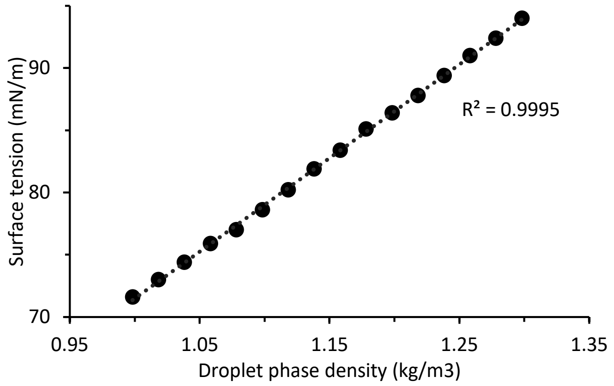

Figure S2. Surface tension as shown by the drop tensiometer as a function of the set droplet phase density of a pendant drop with MilliQ water. A linear trendline is plotted through the points.

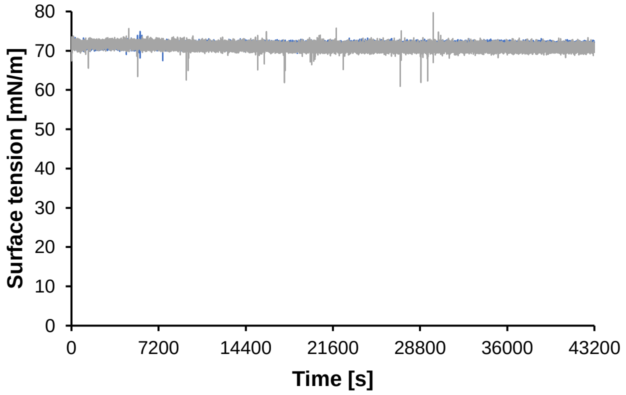

Figure S3. Surface tension as a function of time for milliQ (blue) and 0.2% glass beads (grey). The curves were analysed using the rising drop method. The blue and grey line are overlapping.

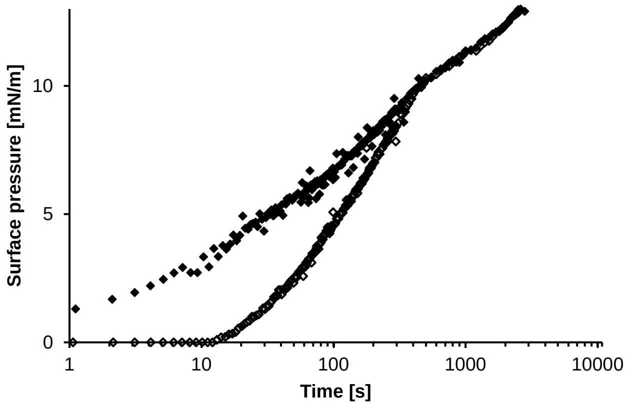

Figure S4. Surface pressure as a function of time for 0.1% WPI(◇) and 0.1% WPI with NaCl (conductivity equal to 1% glass beads; ◆)

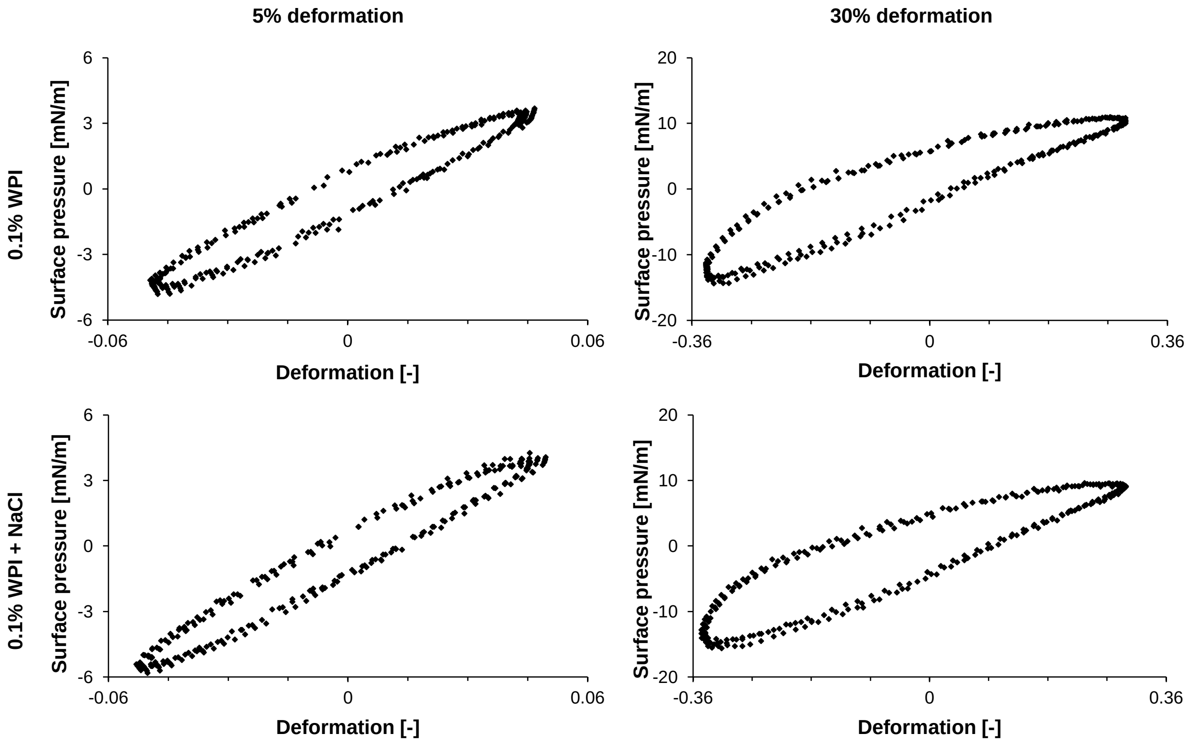

Figure S5. Lissajous plot of 0.1% WPI without and with additional NaCl (conductivity equal to 1% Cospheric P2011SL particles) for 5 and 30% deformation. A representative Lissajous plot is shown for each sample, and comparable plots were obtained from triplicate measurements.

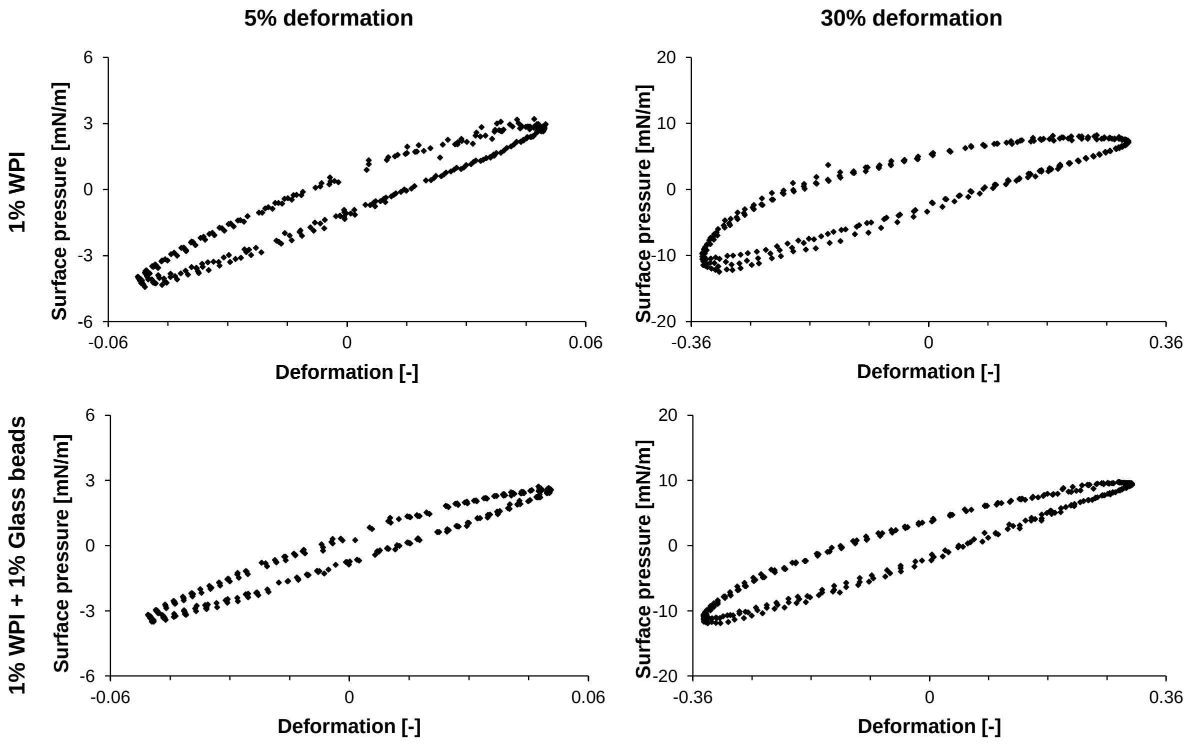

Figure S6. Lissajous plot of 1% WPI with 0 and 1% glass beads for 5 and 30% deformation. A representative Lissajous plot is shown for each sample, and comparable plots were obtained from triplicate measurements.
